# Cystic Fibrosis Mice Are Highly Susceptible to Repeated Acute *Pseudomonas aeruginosa* Pneumonia after Intranasal Inoculation

**DOI:** 10.1155/2024/4769779

**Published:** 2024-02-05

**Authors:** Mariel Manzor, Sophia Koutsogiannaki, Marco DiBlasi, Matthew Schaefers, Gregory Priebe, Koichi Yuki

**Affiliations:** ^1^Department of Anesthesiology, Critical Care and Pain Medicine, Cardiac Anesthesia Division, Boston Children's Hospital, Boston, USA; ^2^Department of Anaesthesia, Harvard Medical School, Boston, USA; ^3^Department of Immunology, Harvard Medical School, Boston, USA; ^4^Broad Institute of MIT and Harvard, Cambridge, USA; ^5^Department of Anesthesiology, Critical Care and Pain Medicine, Critical Care Division, Boston Children's Hospital, Boston, USA; ^6^Department of Pediatrics, Division of Infectious Diseases, Boston Children's Hospital, Boston, USA

## Abstract

Cystic fibrosis (CF) is a genetic disorder caused by mutations in the cystic fibrosis transmembrane conductance regulator (CFTR) that controls chloride current. A number of different CFTR transgenic mouse lines have been developed and subjected to both acute and chronic infection models. However, prior studies showed no substantial differences in bacterial clearance between CF and non-CF mice after single inoculations. Here, using F508del transgenic CF mice, we examined the role of repeated acute *Pseudomonas aeruginosa* (PA) infection, with the second inoculation 7 days after the first. We found that CF mice were more susceptible to PA infection than non-CF mice following the second inoculation, with non-CF mice showing better neutrophil recruitment and effector functions. We further investigated the characteristics of lung immune cells using single-cell RNA sequencing, finding that non-CF lung neutrophils had more prominent upregulation of adhesion molecules including intercellular adhesion molecule-1 (ICAM-1) compared to CF lung neutrophils. Although people with CF are often colonized with bacteria and have high numbers of neutrophils in the airways during chronic infection, these data suggest that CF neutrophils have deficient effector functions in the setting of repeated acute infection.

## 1. Introduction

Cystic fibrosis (CF) is a genetic disorder caused by mutations in the gene encoding the cystic fibrosis transmembrane conductance regulator (CFTR) epithelial chloride channel [1]. Progressive pulmonary disease due to chronic infection and inflammation is the major cause of morbidity and mortality among people with CF [2]. Despite the marked improvement in lung function and overall health of people with CF in the era of CFTR modulator therapies, chronic lung infection persists, albeit with somewhat lower bacterial loads [3]. Transgenic CF KO mice were first developed to study CFTR function and CF, but they suffered from high mortality [4]. Other groups developed the so-called “gut-corrected” CF mouse strain which has a stop codon in the murine *cftr* gene (S489X) but also expresses human CFTR in the gut epithelium due to transgenic introduction of human *Cftr* under the control of the fatty acid binding protein promoter [5]. In other approaches, the F508 deletion mutation (F508del), which is the most commonly seen in people with CF, was introduced into the murine *cftr* gene with a lower mortality [6]. *Pseudomonas aeruginosa* (PA) is a major microbe that infects CF patients. Lipopolysaccharide- (LPS-) rough mucoid PA strains are seen in chronic CF infection, but the initial colonizing PA strains in people with CF are LPS-smooth and nonmucoid [7]. LPS-rough strains lack the serogroup-determining O antigen polysaccharide (also known as O side chain) attached to the LPS core. Acute infection murine models induced by a one-time infection of PA have been reported, but there was no difference in lung bacterial colony forming units (CFUs) between CF and non-CF mice in most studies [8, 9], although some did report increased PA loads in the lungs of CF mice after single inoculations with less internalization of PA in epithelial cells in the CF mice when LPS-smooth strains of PA were inoculated. In the case of LPS-rough mucoid strains, no difference was observed [10].

Chronic infection models have been reported as models to show CF susceptibility to PA infection. Coleman et al. provided PA-containing drinking water to the gut-corrected CF mice for one week and found more oropharyngeal colonization in CF mice [11]. Paroni et al. injected PA-embedded agar bead intratracheally [12]. In this case, beads may serve as a platform for biofilm type of infection. However, there was no difference in CFU in the lungs between CF and non-CF strains.

Here, we hypothesized that repeated introduction of the same colonizing bacterial strain into the lower airways via aspiration likely leads to the transition to defective bacterial clearance on repeated aspiration. Thus, we examined if repeated instillation of PA would be associated with less bacterial clearance and/or altered host response in CF mice.

## 2. Methods

### 2.1. Mouse

F508del mice (Cftr^tm1/Eur^) in the FVB background were provided as breeding pairs by Dr. Bob Scholte (Erasmus University Medical Center, Rotterdam, The Netherlands) and housed under specific pathogen-free condition, with 12-hour light and draft cycles [6, 13, 14]. Mice were given acidified water (sodium acetate, pH 4) to prevent acquisition of PA from the drinking water. All animal protocols were approved by the Institutional Animal Care and Use Committee (IACUC) at Boston Children's Hospital.

### 2.2. Intranasal Instillation of *Pseudomonas aeruginosa* and Bacterial Load Analysis in the Lungs

All the experimental procedures complied with Animal Research Reporting of *In Vivo* Experiment (ARRIVE) guidelines regarding the use of animals in research [15] and the National Institutes of Health guideline for the care and use of laboratory animals and were approved by Boston Children's Hospital animal care and use committee. *Pseudomonas aeruginosa* strain PAK (an LPS-smooth nonmucoid strain) [16] was administered intranasally (dose of 2.5 × 10^6^ CFU in 20 *μ*L) to mice anesthetized by intraperitoneal injection of ketamine/xylazine. Mice (both males and females) were infected at 8 weeks of age. At six hours after the instillation, mice were euthanized, and lungs were explanted and homogenized (PAx1 group). This time point was used to evaluate the role of innate immune responses as we previously performed [17]. Lung homogenates were serially diluted and plated on tryptic soy (TS) agar and incubated overnight for colony forming unit (CFU) counting. A group of mice received repeated intranasal instillation a week later. Similarly, these mice were euthanized at six hours after the second instillation (PAx2 group), and lungs were subjected to bacterial load analysis as above.

### 2.3. Flow Cytometry Analysis

Leukocyte counts in the lungs were analyzed using flow cytometry at time points described in the corresponding figure legend. Lungs were explanted following intravascular perfusion of PBS. Whole lungs were subjected to lung cell isolation. Briefly, lungs were digested with collagenase D and DNase I, and red blood cells were subjected to lysis using low and high osmotic NaCl. Then, cells were stained with Ly6G (1A8) for neutrophils, F4/18 (BM8) for macrophages, NK1.1 (PK136) for NK cells, CD4 (RM4-5) for CD4 cells, CD8 (53-6.7) for CD8 cells, and B220 (RA3-6B2) for B cells. Antibodies were purchased from BioLegend (San Diego, CA). BD Accuri C6 (BD Biosciences, Franklin Lakes, NJ) was used for flow cytometry analysis.

### 2.4. Histology

Lungs were fixed using 4% paraformaldehyde for histological analysis and subjected to hematoxylin and eosin (H&E) staining.

### 2.5. Reverse Transcription and Real-Time Quantitative Polymerase Chain Reaction

Following harvest at time points described in the corresponding figure legend, lungs were snap frozen and stored at -80°C until use. Total RNA was extracted using TRIzol reagent (Life Technologies) per the company's protocol. 1 *μ*g of RNA was subjected to reverse transcription using SuperScript III RNase reverse transcriptase (Life Technologies, San Diego, CA) to create cDNA. Real-time quantitative polymerase chain reaction (PCR) was performed using SYBR green master mix (Applied Biosystems, San Francisco, CA). We measured tumor necrosis factor- (TNF-) *α*, interleukin- (IL-) 1*β*, IL-6, IL-10, IL-12p35, IL-12p40, interferon- (IFN-) *γ*, and keratinocyte chemoattractant (KC). Glyceraldehyde-3-phosphate dehydrogenase (GAPDH) was used as a housekeeping gene. The sequences of primers are listed in Supplementary Table 1.

### 2.6. Measurement of Lung TNF-*α* and IL-6 Levels

Lungs were subjected to homogenization in lysis buffer. Then, supernatants were collected for TNF-*α* and IL-6 level measurement using ELISA kit per company protocol (R&D Systems, Minneapolis, MN).

### 2.7. Neutrophil Phagocytic Function Analysis

Neutrophil phagocytosis was examined using Phagotest kit (Glycotope Biotechnology, Heidelberg, Germany). Lung cells were incubated in complete RPMI 1640 on ice for 10 min, followed by the addition of FITC-labeled *E. coli*. Then, neutrophil suspension was kept on ice as cold control or was placed into 37°C water bath for 20 min. At the end of incubation, cells were transferred back on ice, quenched with ethylene blue, and washed with PBS. Neutrophils were probed with Ly6G. Cells were suspended in PBS/1% PFA and measured by flow cytometry using BD Accuri C6 (BD Biosciences).

### 2.8. Neutrophil Reactive Oxygen Species Formation Analysis

Lung cells were cultured in complete RPMI 1640 at 37°C for 30 min. Dihydrorhodamine 123 (1 *μ*M; Sigma-Aldrich, St. Louis, MO) was added for 5 min at 37°C to lung cells. After washing with RPMI 1640, phorbol 12-myristate 13-acetate (PMA, 100 nM) was added, and the cells was incubated for additional 15 min at 37°C. After one wash, the cells were resuspended in cold PBS with 1% FBS. Neutrophils were probed with Ly6G. Reactive oxygen species (ROS) formation was detected on BD Accuri C6 (BD Biosciences).

### 2.9. Single-Cell RNA Sequencing

#### 2.9.1. 3′ RNA Library Preparation and Sequencing

We performed as we previously described [18]. In brief, following fast thaw in 37°C water bath, cell viability was checked using Thermo Fisher Countess II FL with NucBlue staining. Following cell viability counting, all samples below 70% viability were subjected to dead cell removal via Annexin V bead conjugation to improve quality. Single-cell RNA libraries were generated using the Chromium Single-Cell 3′ kit (10x Genomics, Pleasanton, CA). The cells were counted and loaded onto the Chromium Controller. Loading was performed to target capture of ~3000 gel-in emulsions (GEMs) per sample for downstream analysis, and samples were processed through the Chromium Controller following the standard manufacturer's specifications.

The sequencing libraries were evaluated for quality on the Agilent TapeStation (Agilent Technologies, Palo Alto, CA), and quantified using Qubit 2.0 Fluorometer (Invitrogen, Carlsbad, CA). Pooled libraries were quantified using qPCR (Applied Biosystems) prior to loading onto an Illumina sequencing platform. The samples were sequenced at a configuration compatible with the recommended guidelines as outlined by 10x Genomics.

Raw sequence data (.bcl files) were converted into fastq files and demultiplexed using the 10x Genomics' cellranger mkfastq command. Subsequent UMI and cell barcode deconvolution along with mapping to respective genome were performed using 10x Genomics' Cell Ranger software package to generate the final digital gene expression matrices and cloupe files.

### 2.10. Sequenced Data Analysis

Cloupe files were merged using 10x cloud analysis system. Cell populations were annotated using canonical markers S100A8 and S100A9 (neutrophils); Ear2 and Mrc1 (macrophages); CD3d (T cells); CD79a, CD79b, Pax5, and Ms4a1 (B cells); and F3, Lamb3, and Vim (alveolar type 1/2 cells; AT cells). Differentially expressed genes (DEGs) were defined by log_2_ > 1 and *p* < 0.05. Up- and downregulated DEGs were identified and subjected to KEGG pathway analysis using the Database for Annotation, Visualization, and Integrated Discovery (DAVID) [19, 20].

### 2.11. Statistical Analysis

Statistical analysis was performed using PRISM9 software (GraphPad, La Jolla, CA). Detailed statistical analyses are described in the corresponding figure legends. Statistical significance was defined as *p* < 0.05.

## 3. Results

### 3.1. CF Mice Showed Susceptibility to Acute *Pseudomonas aeruginosa* Infection following Repeated Instillation

**Figure 1:**
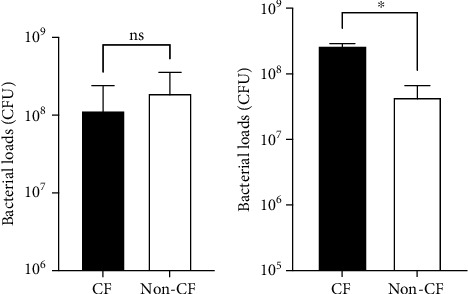
Lung bacterial loads following *Pseudomonas aeruginosa* intranasal instillation. (a) CF and non-CF mice were anesthetized and subjected to intranasal instillation of *Pseudomonas aeruginosa* (PA) strain PAK (2.5 × 10^6^ CFU). At six hours after the instillation, mice were euthanized, and the lungs were removed to assess lung PA loads. Data are shown as mean ± S.D. (*n* = 6 per group) in log_10_ scale. Student *t* test was performed on log_10_-transformed CFU values. ns = not significant. (b) CF and non-CF mice were subjected to intranasal instillation of PA strain PAK (2.5 × 10^6^ CFU) with seven days apart. At six hours after the second instillation, mice were euthanized, and the lungs were removed to assess lung PA loads. Data are shown as mean ± S.D. (*n* = 6 per group) in log_10_ scale. Student *t* test was performed on log_10_ transformed CFU values. ^∗^*p* < 0.05.

We first examined bacterial loads in the lungs at 6 hours after the first intranasal instillation. However, we did not see any significant difference in PA loads between CF and non-CF lungs (Figure 1(a)). However, at 6 hours after repeated instillation of PA, CF mice had higher bacterial loads in the lungs (Figure 1(b)), suggesting that CF mice were more susceptible to repeated PA infection.

### 3.2. CF Mice Showed Less Robust Neutrophil Recruitment after the Second PA Instillation Compared to Non-CF Mice

**Figure 2:**
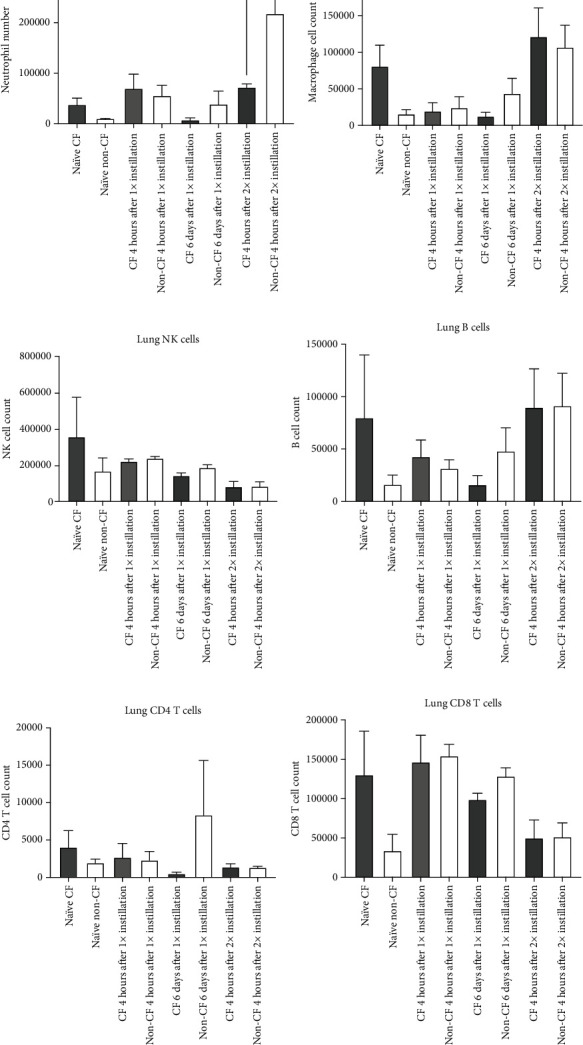
Lung leukocyte counts in CF and non-CF mice. Lung leukocyte counts were examined at baseline, at 4 hours after the one-time instillation, at 6 days after the instillation, and at 4 hours after the second instillation of *Pseudomonas aeruginosa* (PA) in CF and non-CF mice using flow cytometry analysis. (a) Neutrophils, (b) macrophages, (c) NK cells, (d) B cells, (e) CD4 T cells, and (f) CD8 T cells were probed using Ly6G, F4/80, NK1.1, B220, CD4, and CD8 antibodies, respectively. Data are shown as mean ± S.D. (*n* = 3 per arm). One-way ANOVA with the Bonferroni post hoc analysis was performed. ^∗^*p* < 0.05.

**Figure 3:**
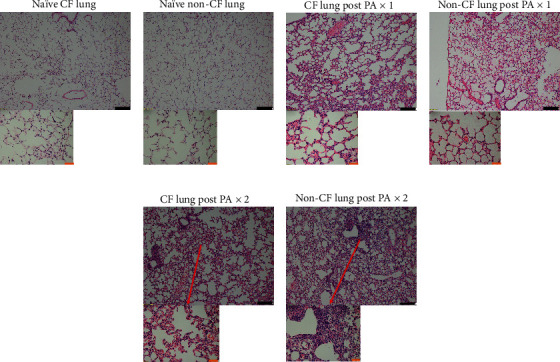
Histological analysis of lungs in CF and non-CF mice. H&E-stained sections from (a) naïve CF lung, (b) naïve non-CF lung, (c) CF lung post one-time instillation (PAx1, at 4 hours after the instillation), (d) non-CF lung post one-time instillation (PAx1, at 4 hours after the instillation), (e) CF lung post two-time instillation (PAx2, at 4 hours after the second instillation), and (f) non-CF lung post two-time instillation (PAx2, at 4 hours after the second instillation). Black bar represents 100 *μ*m, and brown bar represents 20 *μ*m. More leukocyte infiltration was observed in non-CF lung after the second instillation (f).

To understand the underlying mechanism of how CF mice were more susceptible to repeated PA infection, we examined leukocyte subset numbers in the lungs. We did not find any significant difference in the number of macrophages, NK cells, CD4 T cells, CD8 T cells, or B cells between CF and non-CF mice at various time points (Figures 2(b)–2(f)). However, neutrophil numbers were significantly higher in non-CF mice after the second instillation (Figure 2(a)). This finding was compatible with our finding from the histological analysis (Figures 3(a)–3(f)). H&E-stained lung specimens after the second instillation showed more neutrophil infiltration in the lungs of non-CF mice (Figure 3(f)) compared to CF lung.

### 3.3. CF Lungs Showed More Proinflammatory Profiles after the Second Instillation

**Figure 4:**
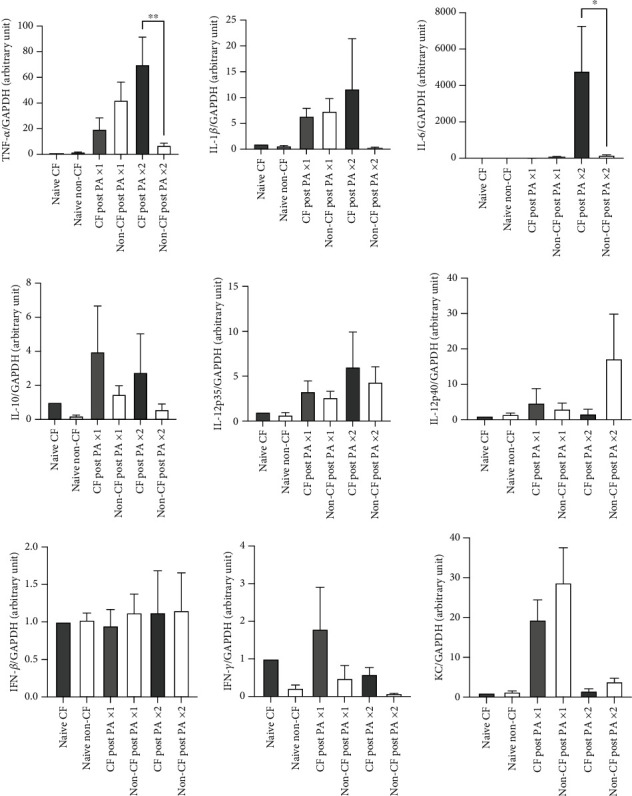
Lung cytokine transcript profiles in CF and non-CF mice. We performed real-time qPCR analysis of a set of cytokine transcripts of the lungs from CF and non-CF mice at baseline, at 4 hours after the one-time instillation, at 6 days after the one-time, and at 4 hours after the second instillation of PA. Data are shown as mean ± S.D. (*n* = 4). Statistical analysis was done using one-way ANOVA with Bonferroni *post hoc* analysis. ^∗∗^*p* < 0.01.

To understand inflammatory status, we examined mRNA expression of a group of cytokines. We found that TNF-*α* and IL-6 mRNA levels were significantly elevated in CF lungs after the second instillation (Figure 4). This may be due to higher bacterial loads in CF lungs following the second instillation. We also determined the protein levels of TNF-*α* and IL-6 using lung lysates. In line with the mRNA findings, TNF-*α* and IL-6 levels were higher in the lungs of CF mice following the second instillation (Suppl. Figure 1).

### 3.4. Non-CF Lung Neutrophils Showed Better Phagocytic Function following Repeated Instillation

**Figure 5:**
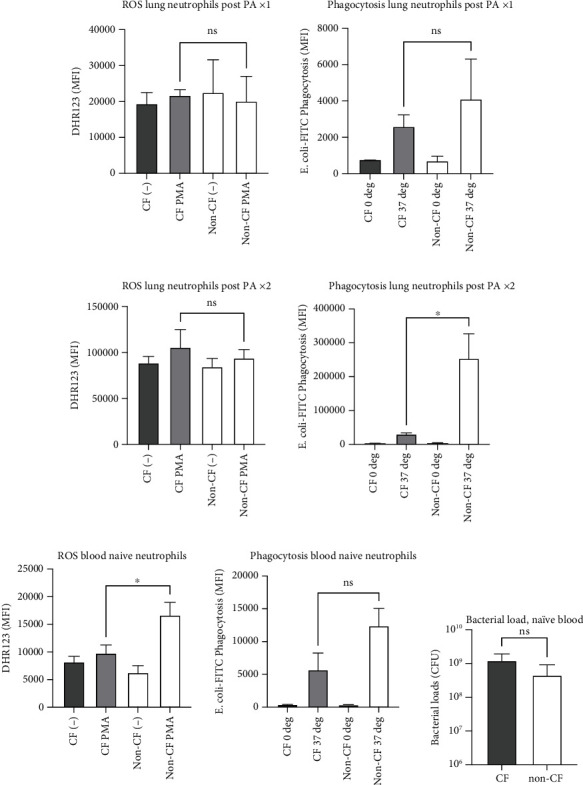
Neutrophil effector functions in CF and non-CF mice. Reactive oxygen species (ROS) formation and phagocytosis were examined as neutrophil effector functions in (a) lung neutrophils post one-time PA instillation (PAx1), (b) lung neutrophils post two-time instillation (PAx2), and (c) blood naïve neutrophils. Data are presented as mean ± S.D. (*n* = 3 per group). One-way ANOVA with Bonferroni *post hoc* analysis was performed to examine statistical significance. ns = not significant. ^∗^*p* < 0.05. (d) The role of blood in bacterial killing was examined by coincubating 100 *μ*L blood and 1 × 10^9^ CFU PA for four hours. Data are shown as mean ± S.D. (*n* = 6 per group) in log scale. Student *t* test was performed on log_10_-transformed CFU values. ns = not significant.

There were more neutrophils in the lungs of non-CF mice after the second instillation. We examined lung neutrophil phagocytic function and ROS production. We did not see any differences in ROS production and phagocytic function after the first instillation between CF and non-CF mice (Figure 5(a)). However, we found that phagocytic function was significantly enhanced in non-CF lung neutrophils after the second instillation compared to their counterpart (Figure 5(b)). Higher lung neutrophil counts with better phagocytic functions in non-CF mice are in line with our bacterial load results (Figure 1(b)). We also examined neutrophil effector functions in naïve mice. We found that ROS was enhanced in non-CF naïve neutrophils compared to CF naïve neutrophils, but not phagocytosis (Figure 5(c)). Difference in neutrophil effector function profiles among naïve, post one-time instillation, and two-time instillation might have indicated that neutrophil phenotypical changes occurred over time following PA infection. When we examined bacterial loads in naïve blood, we did not see statistical difference although there was a trend that the average CFU was higher in CF mice (Figure 5(d)). This result is in line with the bacterial load result in the lungs following one-time instillation (Figure 1(a)).

### 3.5. Single-Cell RNA Sequencing Results Show the Upregulation of Adhesion Molecules in Non-CF Neutrophils following Repeated Instillation

To obtain further insights into the function of lung cells, we performed single-cell RNA sequencing analysis of mouse lung cells at baseline as well as after the second instillation.

**Figure 6:**
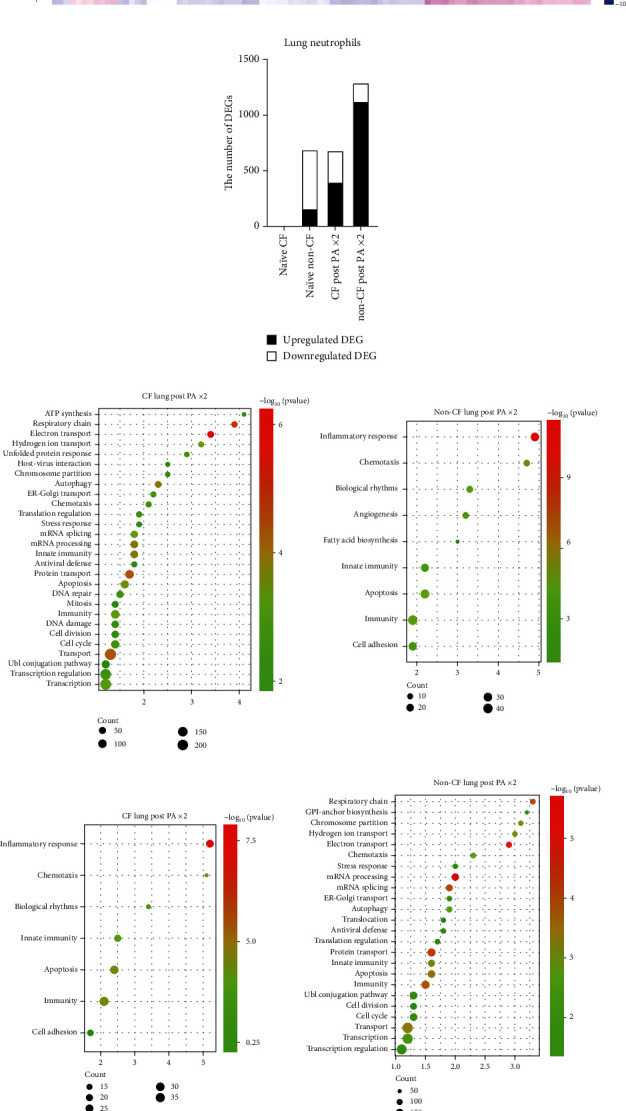
Single-cell RNA sequencing analysis of lung cells and neutrophil DEG/ontology. (a) Naïve CF and non-CF lung cells and CF and non-CF lung cells post two-time instillation were subjected to single-cell RNA sequencing analysis. Annotation results are shown. (b) Heatmap of lung neutrophil differentially expressed genes (DEGs). (c) The number of up- and downregulated DEGs. (d) Upregulated DEGs in CF and non-CF lung neutrophils were subjected to KEGG pathway analysis and presented as bubble enrichment maps. (e) Downregulated DEGs in CF and non-CF lung neutrophils were subjected to KEGG pathway analysis and presented as bubble enrichment maps. (f) Adhesion molecule expression pattern was shown on the heatmap. (g) *tlr4* expression level was shown in violin plots.

We showed cell annotations in Figure 6(a). Neutrophil transcriptomic heatmap is shown in Figure 6(b), suggesting that the post-PAx2 instillation non-CF neutrophil transcriptomic profile was distinct. We determined up- and downregulated differentially expressed genes (DEGs) (Figure 6(c)). We identified most DEGs in non-CF neutrophils post-PAx2 instillation. We also performed ontology analysis. Chemotaxis and apoptosis were annotated from both up- and downregulated DEGs in CF and non-CF mice. In contrast, cell adhesion ontology was upregulated only in non-CF lung neutrophils following repeated instillation and downregulated in CF lung neutrophils (Figures 6(d) and 6(e)). Adhesion molecule expression profile was shown in the heatmap, demonstrating that they were highly upregulated only in non-CF neutrophils post-PAx2 instillation (Figure 2(f)). For example, *ICAM-1* is highly involved in neutrophil effector functions. Since intercellular adhesion molecule-1 (ICAM-1) expressing neutrophils exhibit enhanced phagocytosis [21], this result is in line with the increased phagocytosis we observed in non-CF lung neutrophils. LPS on PA primarily targets Toll-like receptor 4 (TLR4), which would induce ICAM-1 expression [21, 22]. As shown, TLR4 expression was higher in non-CF lung neutrophils following repeated PA exposure (Figure 6(g)).

**Figure 7:**
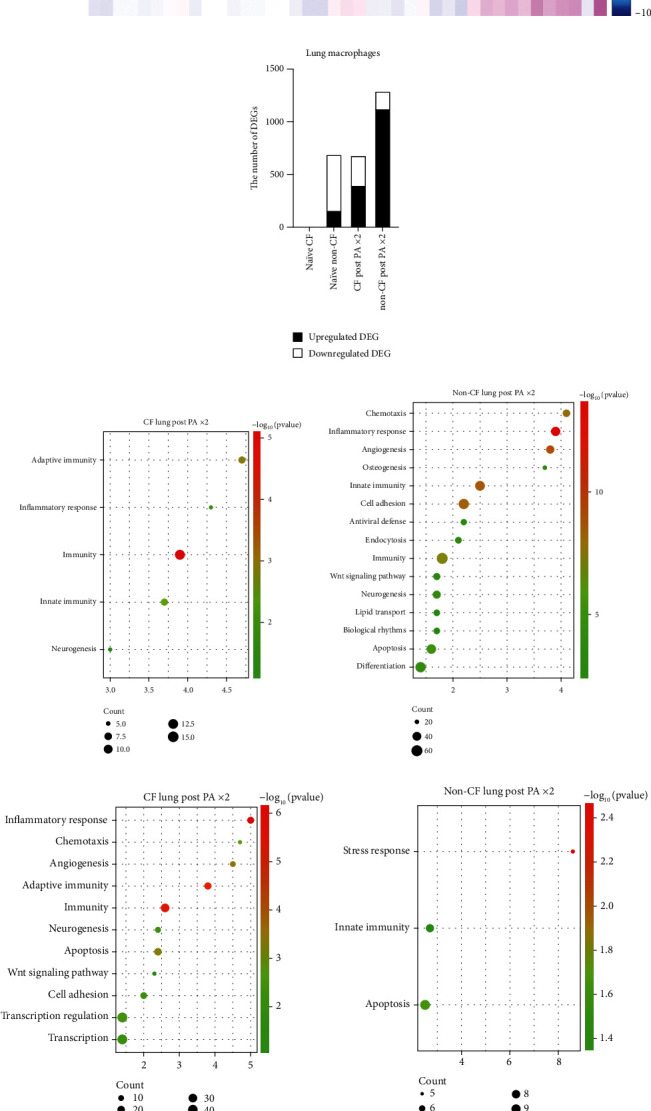
Single-cell RNA sequencing analysis of lung macrophage cells. (a) Heatmap of lung macrophage differentially expressed genes (DEGs). (b) The number of up- and downregulated DEGs. (c) Upregulated DEGs in CF and non-CF lung macrophages were subjected to KEGG pathway analysis and presented as bubble enrichment maps. (d) Downregulated DEGs in CF and non-CF lung macrophages were subjected to KEGG pathway analysis and presented as bubble enrichment maps.

We also examined transcriptomic profiles of other cell types. The heatmap for macrophages is included in Figure 7(a). We also determined up- and downregulated DEGs in macrophages (Figure 7(b)). As in the case of neutrophils, the number of DEGs was highest in non-CF macrophages post-PA instillation. Non-CF macrophages showed an upregulation of chemotaxis and cell adhesion (Figure 7(c)), while they were downregulated in CF macrophages (Figure 7(d)). However, we did not see any difference in lung macrophage number between CF and non-CF mice (Figure 2(b)). Apoptosis was downregulated in CF macrophages (Figure 7(d)), which may explain macrophage counts in the lungs. Although NK cells showed limited number of DEGs (Suppl Figure 2a), non-CF post-PA NK cells showed more distinct transcriptomic profiles than others on the heatmap (Suppl Figure 2b). Given its very limited number of DEGs, we did not perform ontology analysis for NK cells. We also performed the analysis of B cells (Suppl Figure 3a-3d), T cells (Suppl Figure 4a-4d), and AT cells (Suppl Figure 5) analyses. Based on transcriptomic profiles, there was a significant difference in post-PA instillation non-CF and CF B cells and T cells (Suppl. Fig. 3-4). For example, CF lung B cells showed significant upregulation of cell adhesion (Suppl. Figure 3c). CF lung T cells showed significant upregulation of apoptosis, mitosis, cell division, and cell cycling (Suppl. Figure 4c). CF lung AT cells showed significant upregulation of apoptosis and downregulation of inflammatory responses and immunity (Suppl. Figure 5c-5d).

## 4. Discussion

Here, we describe that repeated acute PA exposure worsened PA bacterial loads in the lungs of CF mice compared to non-CF mice, which was associated with less neutrophil recruitment and effector functions.

Earlier *cftr* KO mouse strains were developed by various investigators to recapitulate CF pathophysiology. However, their use was significantly hindered by high mortality rates associated with intestinal obstruction [4], which is less prevalent in mice homozygous for the F508 deletion mutation in the *cftr* gene [6]. Infection models with CF mice are divided into two categories, acute and chronic infection models. Acute lung infection models have been established by a one-time infection with PA. However, the clinical course of the acute PA lung infection was, in most studies, indistinguishable between CF and non-CF mice that share the same background [8–10]. An artificial bead model has been used to mimic bacterial persistence in lungs, but the model is also invasive since most investigators administer the bacteria via intratracheal instillation. In addition, no difference in lung CFUs was observed between CF and non-CF mice using the model. Acute lung infection models, however, are informative to understand how CF responds to acute infection. Clinically, people with CF succumb to chronic lung infection, which is likely initially established by repeated aspiration of colonizing bacteria from the oropharynx into the lower airways. We found that CF mice were more susceptible to the second infection, which was associated with a lower number of recruited neutrophils to the lungs as well as defective phagocytic function.

The *Cftr* gene is highly expressed in barrier tissues such as lung epithelial cells and gut epithelial cells [23]. The loss of CFTR function leads to an altered ion flux through the airway epithelium and ablation of mucociliary clearance. This is considered one mechanism responsible for the colonization of bacteria in the CF airway. In addition to epithelial cells, CFTR expression in phagocytes has been reported. Neutrophils express CFTR in the plasma membrane and in intracellular vesicles related to phagosomes [24]. Although chloride current is important in neutrophil physiology [25], neutrophils have chloride channels other than CFTR such as the glycine receptor [26] and voltage-gated chloride channel [27]. In fact, the role of CFTR in neutrophil functions has been conflicting in the literature. For example, ROS was attenuated in human CF neutrophils in some reports [24, 28, 29], but not in others [30–32]. Similarly, neutrophil phagocytosis was impaired in CF patients in one study [33], but not in another [34]. In the current study, we have examined murine lung neutrophil ROS and phagocytosis after the first and second instillation of PA. Phagocytosis was significantly impaired only after the second instillation. These changes might have been due to the inflammatory milieu. In fact, TNF-*α* and IL-6 levels were significantly elevated in the CF lungs after the second instillation compared to non-CF lungs. Ontology analysis showed upregulated adhesion molecules including *ICAM-1* in non-CF lung neutrophils after PA instillation. We and others showed that ICAM-1 is induced in neutrophils via LPS stimulation [21, 22]. TLR4, which senses LPS, was also upregulated in non-CF lung neutrophils after PA instillation. Because CF mice had higher bacterial loads in the lungs after the second instillation, whether this was driven by more available LPS in CF mice or CFTR affected intrinsic TLR4 signaling pathway needs to be determined. This will be a subject of future investigation.

One of the features of the CF lung is the accumulation of neutrophils. This is considered due to chronic colonization of bacteria. However, we did not extend our study to more chronic phases of colonization. Rather, we primarily examined how CF mice responded to repeated acute PA infection. We found that CF mice become more susceptible to repeated acute PA infection associated with less recruitment of neutrophils along with less neutrophil effector functions. Although CF lungs may harbor many neutrophils in a chronically colonized state, our finding indicates that they may not have proper neutrophil recruitment when they experience repeated acute infection. In addition to neutrophils, we also examined the phenotype of other leukocytes. CF lung macrophages showed downregulation of chemotaxis and adhesion. CF T cells showed apoptosis and enhanced cell cycle activity. CF B cells showed an increase in cell adhesion. The number of these cell types was comparable between CF and non-CF mice in the lungs, but it would deserve future investigation of functional analysis.

In conclusion, we described a repeated acute PA infection model in transgenic CF mice. Following repeated instillation of PA by the intranasal route, CF lungs were more susceptible to infection. Lower neutrophil numbers/function and less adhesion molecule upregulation are likely responsible for this phenotype, but the underlying regulatory mechanism of adhesion molecule expression in CF lung neutrophils needs further investigation.

## Data Availability

Upon request, we would provide all the data available.

## Supplementary Materials

Supplementary 1 Supplementary Figure 1: TNF-*α* and IL-6 levels in the lungs. CF and non-CF mice were subjected to PA instillation twice. At 6 hours after the second instillation, lungs were obtained and subjected to homogenization followed by TNF-*α* and IL-6 measurement. Data were shown as mean ± S.D. of 4 samples per group. One-way ANOVA with the Bonferroni *post hoc* analysis was performed. ^∗∗∗^*p* < 0.001. Supplementary Figure 2: single-cell RNA sequencing analysis of lung NK cells. (a) Heatmap of lung NK cell differentially expressed genes (DEGs). (b) The number of up- and downregulated DEGs. Supplementary Figure 3: single-cell RNA sequencing analysis of lung B cells. (a) Heatmap of lung B cell differentially expressed genes (DEGs). (b) The number of up- and downregulated DEGs. (c) Upregulated DEGs in CF and non-CF lung B cells were subjected to KEGG pathway analysis and presented as bubble enrichment maps. (d) Downregulated DEGs in CF and non-CF lung B cells were subjected to KEGG pathway analysis and presented as bubble enrichment maps. Supplementary Figure 4: single-cell RNA sequencing analysis of lung T cells. (a) Heatmap of lung T cell differentially expressed genes (DEGs). (b) The number of up- and downregulated DEGs. (c) Upregulated DEGs in CF and non-CF lung T cells were subjected to KEGG pathway analysis and presented as bubble enrichment maps. (d) Downregulated DEGs in CF and non-CF lung T cells were subjected to KEGG pathway analysis and presented as bubble enrichment maps. Supplementary Figure 5: single-cell RNA sequencing analysis of lung AT cells. (a) Heatmap of lung AT cell differentially expressed genes (DEGs). (b) The number of up- and downregulated DEGs. (c) Upregulated DEGs in CF and non-CF lung AT cells were subjected to KEGG pathway analysis and presented as bubble enrichment maps. (d) Downregulated DEGs in CF and non-CF lung AT cells were subjected to KEGG pathway analysis and presented as bubble enrichment maps.

Supplementary 2 Supplementary Table 1: primer sequences for RT-qPCR.

## References

[1] Cutting G. R. (2015). Cystic fibrosis genetics: from molecular understanding to clinical application. *Nature Reviews Genetics*.

[2] Ong T., Ramsey B. W. (2023). Cystic fibrosis. *JAMA*.

[3] Nichols D. P., Morgan S. J., Skalland M. (2023). Pharmacologic improvement of CFTR function rapidly decreases sputum pathogen density, but lung infections generally persist. *The Journal of Clinical Investigation*.

[4] McCarron A., Parsons D., Donnelley M. (2021). Animal and cell culture models for cystic fibrosis: which model is right for your application?. *The American Journal of Pathology*.

[5] Zhou L., Dey C. R., Wert S. E., DuVall M. D., Frizzell R. A., Whitsett J. A. (1994). Correction of lethal intestinal defect in a mouse model of cystic fibrosis by human CFTR. *Science*.

[6] Wilke M., Buijs-Offerman R. M., Aarbiou J. (2011). Mouse models of cystic fibrosis: phenotypic analysis and research applications. *Journal of Cystic Fibrosis*.

[7] Hancock R. E., Mutharia L. M., Chan L., Darveau R. P., Speert D. P., Pier G. B. (1983). Pseudomonas aeruginosa isolates from patients with cystic fibrosis: a class of serum-sensitive, nontypable strains deficient in lipopolysaccharide O side chains. *Infection and Immunity*.

[8] Sandri A., Lleo M. M., Signoretto C., Boaretti M., Boschi F. (2021). Protease inhibitors elicit anti-inflammatory effects in CF mice with Pseudomonas aeruginosa acute lung infection. *Clinical and Experimental Immunology*.

[9] Munder A., Wolbeling F., Kerber-Momot T. (2011). Acute intratracheal Pseudomonas aeruginosa infection in cystic fibrosis mice is age-independent. *Respiratory Research*.

[10] Schroeder T. H., Reiniger N., Meluleni G., Grout M., Coleman F. T., Pier G. B. (2001). Transgenic cystic fibrosis mice exhibit reduced early clearance of Pseudomonas aeruginosa from the respiratory tract. *Journal of Immunology*.

[11] Coleman F. T., Mueschenborn S., Meluleni G. (2003). Hypersusceptibility of cystic fibrosis mice to chronic Pseudomonas aeruginosa oropharyngeal colonization and lung infection. *Proceedings of the National Academy of Sciences of the United States of America*.

[12] Paroni M., Moalli F., Nebuloni M. (2013). Response of CFTR-deficient mice to long-term chronic Pseudomonas aeruginosa infection and PTX3 therapy. *The Journal of Infectious Diseases*.

[13] French P. J., van Doorninck J. H., Peters R. H. (1996). A delta F508 mutation in mouse cystic fibrosis transmembrane conductance regulator results in a temperature-sensitive processing defect in vivo. *The Journal of Clinical Investigation*.

[14] van Doorninck J. H., French P. J., Verbeek E. (1995). A mouse model for the cystic fibrosis delta F508 mutation. *The EMBO Journal*.

[15] Kilkenny C., Browne W., Cuthill I. C., Emerson M., Altman D. G. (2010). Animal research: reporting in vivo experiments: the ARRIVE guidelines. *British Journal of Pharmacology*.

[16] Cain A. K., Nolan L. M., Sullivan G. J., Whitchurch C. B., Filloux A., Parkhill J. (2019). Complete genome sequence of Pseudomonas aeruginosa reference strain PAK. *Microbiology Resource Announcements*.

[17] Koutsogiannaki S., Schaefers M. M., Okuno T. (2017). From the cover: prolonged exposure to volatile anesthetic isoflurane worsens the outcome of polymicrobial abdominal sepsis. *Toxicological Sciences*.

[18] Koutsogiannaki S., Kim S., Yuki K. (2023). Age-dependent transcriptomic profiles of leukocytes in pediatric population. *Clinical Immunology*.

[19] Sherman B. T., Hao M., Qiu J. (2022). DAVID: a web server for functional enrichment analysis and functional annotation of gene lists (2021 update). *Nucleic Acids Research*.

[20] Huang D. W., Sherman B. T., Lempicki R. A. (2009). Systematic and integrative analysis of large gene lists using DAVID bioinformatics resources. *Nature Protocols*.

[21] Woodfin A., Beyrau M., Voisin M. B. (2016). ICAM-1-expressing neutrophils exhibit enhanced effector functions in murine models of endotoxemia. *Blood*.

[22] Okuno T., Koutsogiannaki S., Hou L. (2019). Volatile anesthetics isoflurane and sevoflurane directly target and attenuate Toll-like receptor 4 system. *The FASEB Journal*.

[23] Crawford I., Maloney P. C., Zeitlin P. L. (1991). Immunocytochemical localization of the cystic fibrosis gene product CFTR. *Proceedings of the National Academy of Sciences of the United States of America*.

[24] Robledo-Avila F. H., Ruiz-Rosado J. D., Brockman K. L. (2018). Dysregulated calcium homeostasis in cystic fibrosis neutrophils leads to deficient antimicrobial responses. *The Journal of Immunology*.

[25] Menegazzi R., Busetto S., Dri P., Cramer R., Patriarca P. (1996). Chloride ion efflux regulates adherence, spreading, and respiratory burst of neutrophils stimulated by tumor necrosis factor-alpha (TNF) on biologic surfaces. *The Journal of Cell Biology*.

[26] Wheeler M., Stachlewitz R. F., Yamashina S., Ikejima K., Morrow A. L., Thurman R. G. (2000). Glycine-gated chloride channels in neutrophils attenuate calcium influx and superoxide production. *The FASEB Journal*.

[27] Schumann M. A., Raffin T. A. (1994). Activation of a voltage-dependent chloride current in human neutrophils by phorbol 12-myristate 13-acetate and formyl-methionyl-leucyl-phenylalanine. The role of protein kinase C. *Journal of Biological Chemistry*.

[28] Houston N., Stewart N., Smith D. S., Bell S. C., Champion A. C., Reid D. W. (2013). Sputum neutrophils in cystic fibrosis patients display a reduced respiratory burst. *Journal of Cystic Fibrosis*.

[29] Fruhwirth M., Ruedl C., Ellemunter H., Bock G., Wolf H. (1998). Flow–cytometric evaluation of oxidative burst in phagocytic cells of children with cystic fibrosis. *International Archives of Allergy and Immunology*.

[30] Witko-Sarsat V., Allen R. C., Paulais M. (1996). Disturbed myeloperoxidase-dependent activity of neutrophils in cystic fibrosis homozygotes and heterozygotes, and its correction by amiloride. *Journal of Immunology*.

[31] McKeon D. J., Cadwallader K. A., Idris S. (2010). Cystic fibrosis neutrophils have normal intrinsic reactive oxygen species generation. *The European Respiratory Journal*.

[32] Kolpen M., Hansen C. R., Bjarnsholt T. (2010). Polymorphonuclear leucocytes consume oxygen in sputum from chronic Pseudomonas aeruginosa pneumonia in cystic fibrosis. *Thorax*.

[33] Alexis N. E., Muhlebach M. S., Peden D. B., Noah T. L. (2006). Attenuation of host defense function of lung phagocytes in young cystic fibrosis patients. *Journal of Cystic Fibrosis*.

[34] Hayes E., Murphy M. P., Pohl K. (2020). Altered degranulation and pH of neutrophil phagosomes impacts antimicrobial efficiency in cystic fibrosis. *Frontiers in Immunology*.

