# Supplementary material for: Cystic Fibrosis Mice Are Highly Susceptible to Repeated Acute Pseudomonas aeruginosa Pneumonia after Intranasal Inoculation
Source: Biomed Res Int. 2024 Feb 5;2024:4769779. doi: 10.1155/2024/4769779 (PMC10861279; doi:10.1155/2024/4769779)
Supplement: Supplementary 1 — Supplementary Figure 1: TNF-α and IL-6 levels in the lungs. CF and non-CF mice were subjected to PA instillation twice. At 6 hours after the second instillation, lungs were obtained and subjected to homogenization followed by TNF-α and IL-6 measurement. Data were shown as mean ± S.D. of 4 samples per group. One-way ANOVA with the Bonferroni post hoc analysis was performed. ∗∗∗p < 0.001. Supplementary Figure 2: single-cell RNA sequencing analysis of lung NK cells. (a) Heatmap of lung NK cell differentially expressed genes (DEGs). (b) The number of up- and downregulated DEGs. Supplementary Figure 3: single-cell RNA sequencing analysis of lung B cells. (a) Heatmap of lung B cell differentially expressed genes (DEGs). (b) The number of up- and downregulated DEGs. (c) Upregulated DEGs in CF and non-CF lung B cells were subjected to KEGG pathway analysis and presented as bubble enrichment maps. (d) Downregulated DEGs in CF and non-CF lung B cells were subjected to KEGG pathway analysis and presented as bubble enrichment maps. Supplementary Figure 4: single-cell RNA sequencing analysis of lung T cells. (a) Heatmap of lung T cell differentially expressed genes (DEGs). (b) The number of up- and downregulated DEGs. (c) Upregulated DEGs in CF and non-CF lung T cells were subjected to KEGG pathway analysis and presented as bubble enrichment maps. (d) Downregulated DEGs in CF and non-CF lung T cells were subjected to KEGG pathway analysis and presented as bubble enrichment maps. Supplementary Figure 5: single-cell RNA sequencing analysis of lung AT cells. (a) Heatmap of lung AT cell differentially expressed genes (DEGs). (b) The number of up- and downregulated DEGs. (c) Upregulated DEGs in CF and non-CF lung AT cells were subjected to KEGG pathway analysis and presented as bubble enrichment maps. (d) Downregulated DEGs in CF and non-CF lung AT cells were subjected to KEGG pathway analysis and presented as bubble enrichment maps. [file 4769779.f1.pdf]

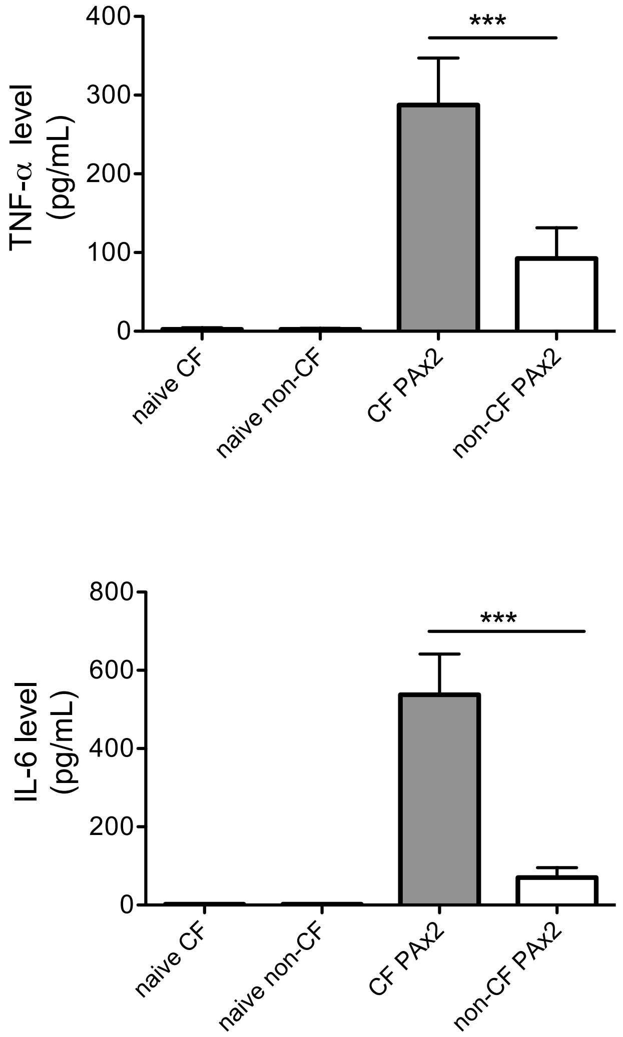

Supplementary Figure 1. TNF-α and IL-6 levels in the lungs. CF and non-CF mice were subjected to PA instillation twice. At 6 hours after the second instillation, lungs were obtained and subjected to homogenization followed by TNF-α and IL-6 measurement. Data were shown as mean +/- S.D. of 4 samples per group. One-way ANOVA with *post hoc* Bonferroni analysis was performed. *** $p < 0.001$.

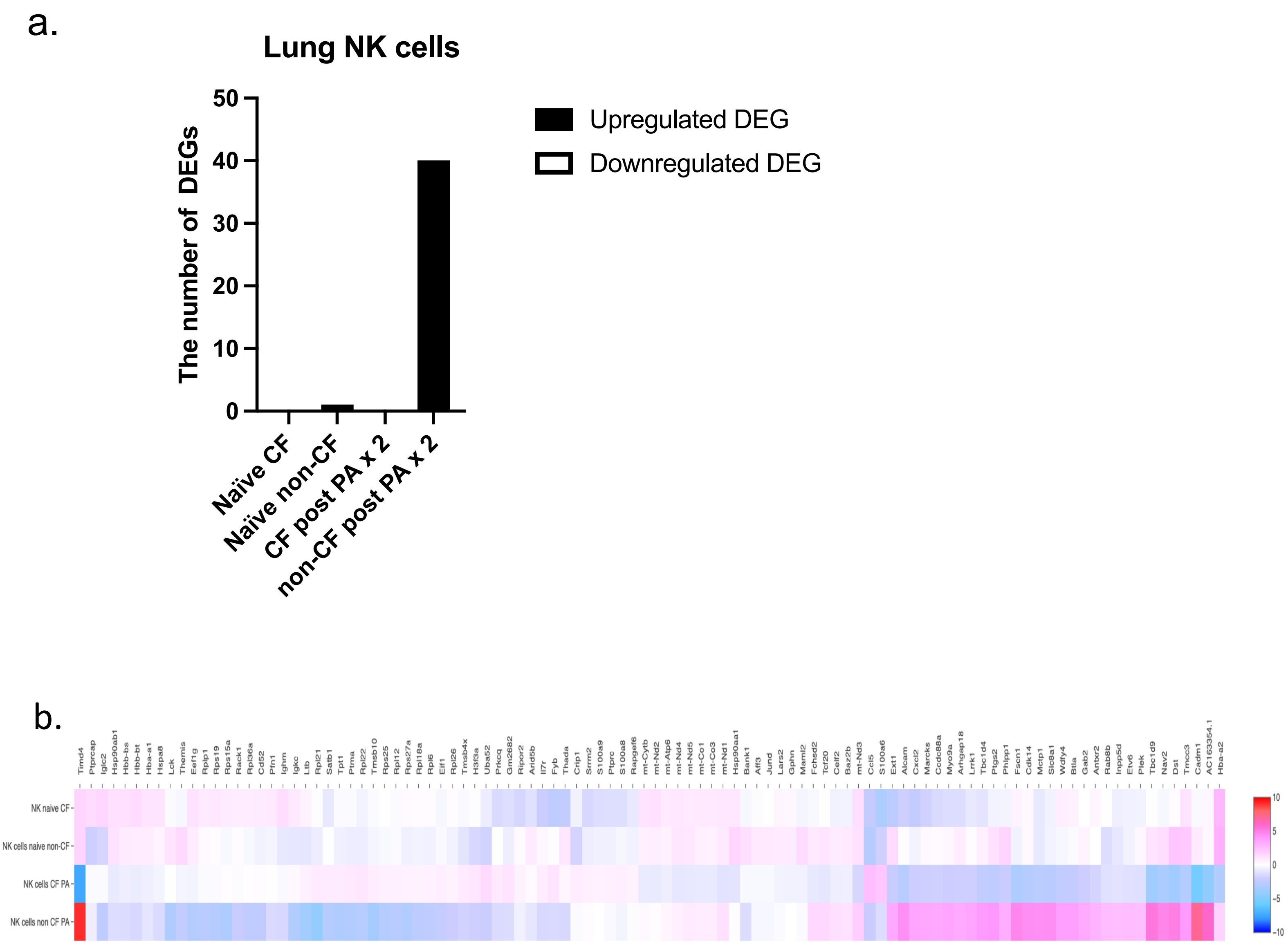

Supplementary Figure 2. Single cell RNA sequencing analysis of lung NK cells

(a) Heatmap of lung NK cell differentially expressed genes (DEGs).

(b) The number of up- and down-regulated DEGs.

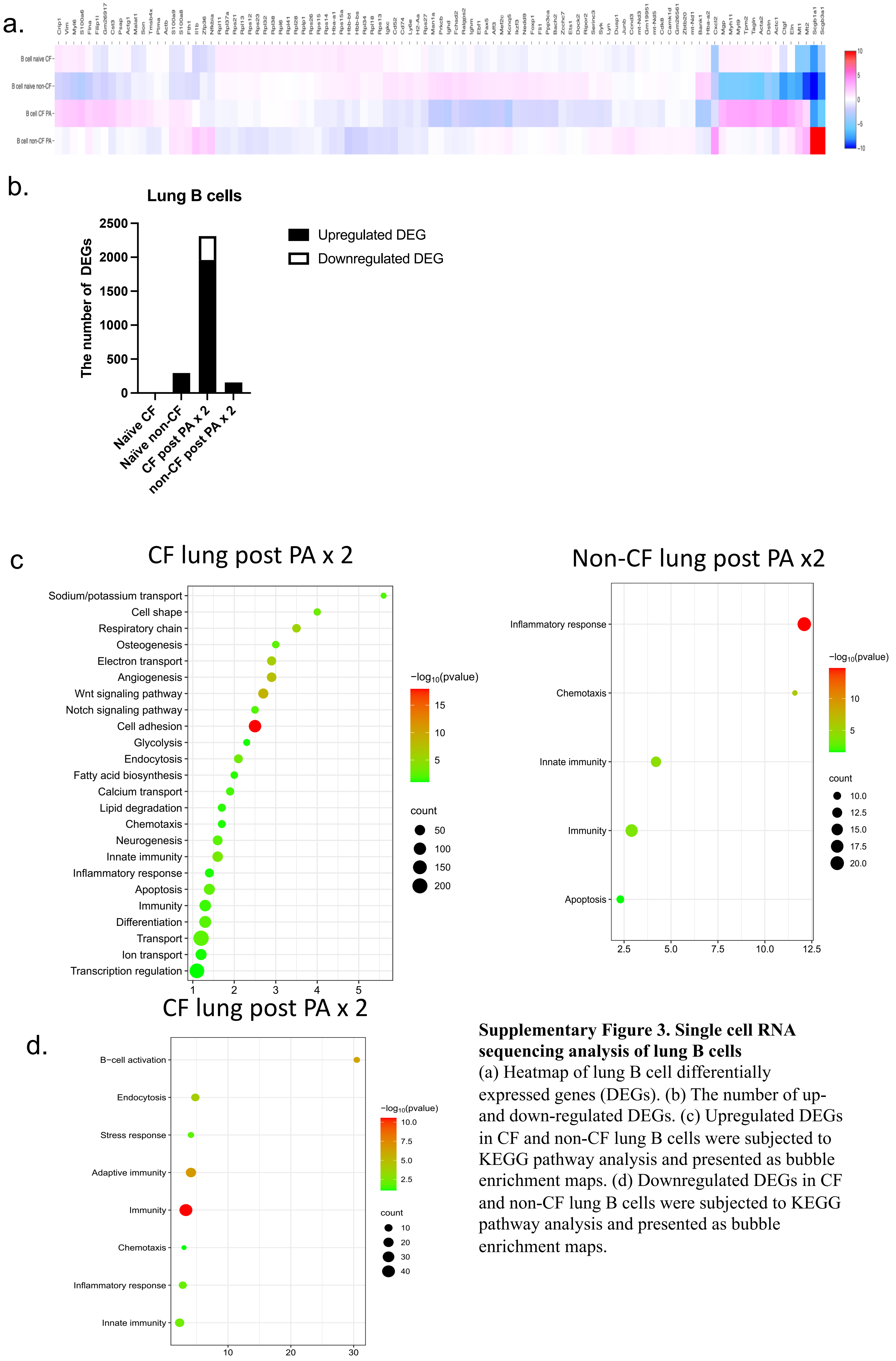

Supplementary Figure 3. Single cell RNA sequencing analysis of lung B cells

(a) Heatmap of lung B cell differentially expressed genes (DEGs). (b) The number of up- and down-regulated DEGs. (c) Upregulated DEGs in CF and non-CF lung B cells were subjected to KEGG pathway analysis and presented as bubble enrichment maps. (d) Downregulated DEGs in CF and non-CF lung B cells were subjected to KEGG pathway analysis and presented as bubble enrichment maps.

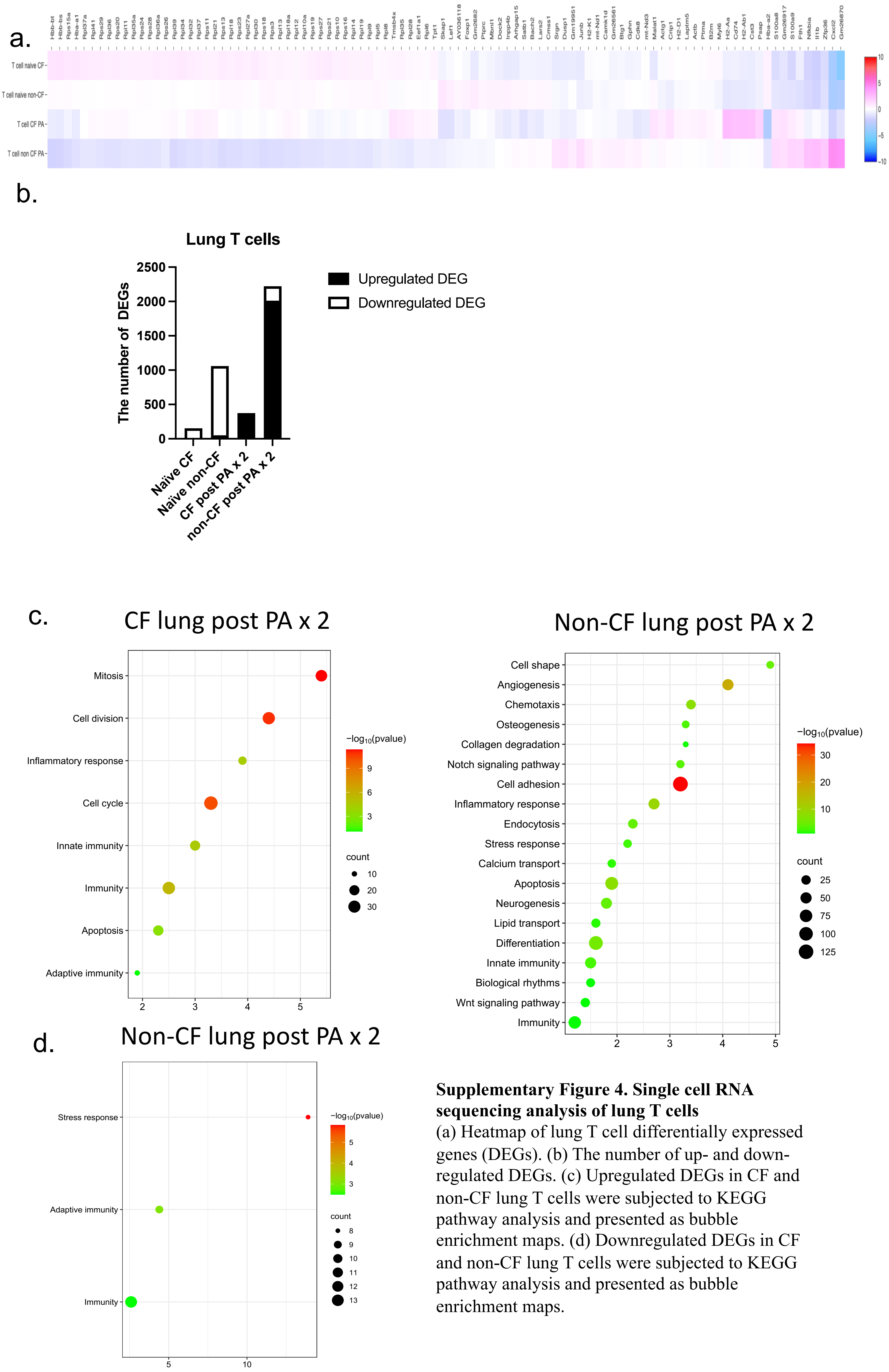

Supplementary Figure 4. Single cell RNA sequencing analysis of lung T cells

(a) Heatmap of lung T cell differentially expressed genes (DEGs). (b) The number of up- and down-regulated DEGs. (c) Upregulated DEGs in CF and non-CF lung T cells were subjected to KEGG pathway analysis and presented as bubble enrichment maps. (d) Downregulated DEGs in CF and non-CF lung T cells were subjected to KEGG pathway analysis and presented as bubble enrichment maps.

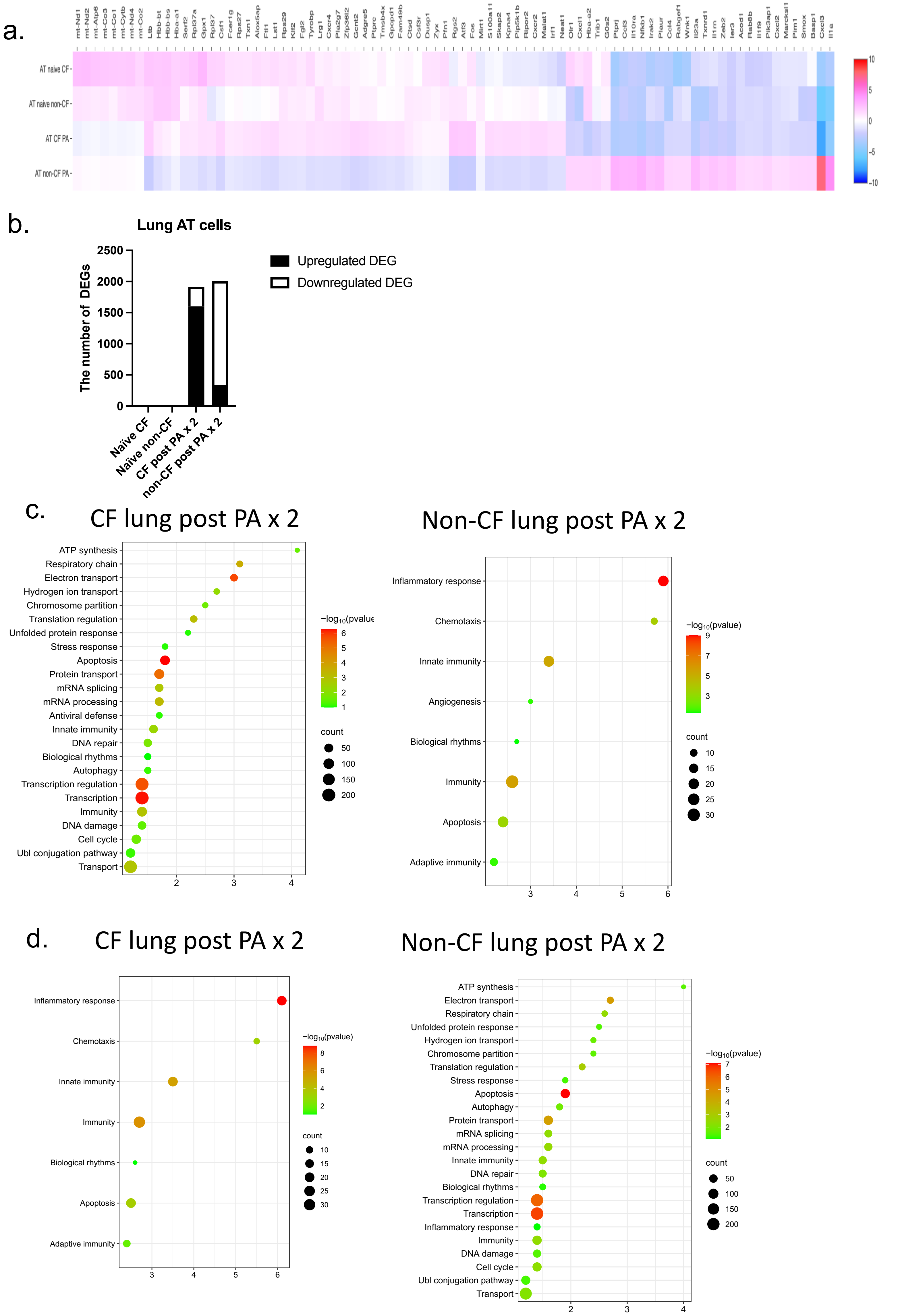

Supplementary Figure 5. Single cell RNA sequencing analysis of lung AT cells
(a) Heatmap of lung AT cell differentially expressed genes (DEGs). (b) The number of up- and down-regulated DEGs. (c) Upregulated DEGs in CF and non-CF lung AT cells were subjected to KEGG pathway analysis and presented as bubble enrichment maps. (d) Downregulated DEGs in CF and non-CF lung AT cells were subjected to KEGG pathway analysis and presented as bubble enrichment maps.
