# Supplementary material for: Cystic Fibrosis Mice Are Highly Susceptible to Repeated Acute Pseudomonas aeruginosa Pneumonia after Intranasal Inoculation
Source: Biomed Res Int. 2024 Feb 5;2024:4769779. doi: 10.1155/2024/4769779 (PMC10861279; doi:10.1155/2024/4769779)
Supplement: Supplementary 2 — Supplementary Table 1: primer sequences for RT-qPCR. [file 4769779.f2.docx]

**Supplementary Table 1**

|  |  |  |
| --- | --- | --- |
| **Gene** | **Forward primer** | **Reverse primer** |
| TNF-α | CTGTAGCCCACGTCGTAGC | TTGAGATCCATGCCGTTG |
| IL-1β | TGTAATGAAAGACGGCACACC | TCTTCTTTGGGTATTGCTTGG |
| IL-6 | GCTACCAAACTGGATATAATCAGGA | CCAGGTAGCTATGGTACTCCAGAA |
| IL-10 | CAGAGCCACATGCTCCTAGA | GTCCAGCTGGTCCTTTGTTT |
| IL-12p35 | CTGTGCCTTGGTAGCATCTATG | GCAGAGTCTCGCCATTATGATTC |
| IL-12p40 | TGGTTTGCCATCGTTTTGCTG | ACAGGTGAGGTTCACTGTTTCT |
| IFN-β | AGGGCGGACTTCAAGATC | CTCATTCCACCCAGTGCT |
| IFN-γ | ATGAACGCTACACACTGCATC | CCATCCTTTTGCCAGTTCCTC |
| KC | CTGGGATTCACCTCAAGAACATC | CAGGGTCAAGGCAAGCCTC |
| GAPDH | GCACAGTCAAGGCCGAGAAT | GCCTTCTCCATGGTGGTGAA |
|  |  |  |
